# Ergonomic risk exposure and work ability among young dental professionals in China: A cross‐sectional study

**DOI:** 10.1002/1348-9585.12154

**Published:** 2020-07-25

**Authors:** Sihao Lin, Zhenyi Wu, Wenjuan Tang, Guoxi Xu, Xianzhe Zeng

**Affiliations:** ^1^ School of Management Putian University Putian China; ^2^ Shanghai Municipal Center for Health Promotion Shanghai China; ^3^ Fujian Center for occupational disease and Chemical Poisoning Control and Prevention Fuzhou China; ^4^ Quanzhou Anke Occupational Health Service Co. Ltd. Quanzhou China

**Keywords:** China, dental professionals, ergonomic exposure, work ability

## Abstract

**Background:**

Exposure to high ergonomic risk resulted in an increasing prevalence of musculoskeletal disorders among dental professional. However, little is known about the high exposure risk impact on work ability among dental professionals.

**Objective:**

We conducted a cross‐sectional study to examine the association between ergonomic risk exposure and work ability among young dental professionals in their early careers.

**Methods:**

A total of 230 dental professionals including dentists, dental assistants, and nurses were clustered sampled from three hospitals in Guangzhou, south of China. We used the Quick Ergonomic Check (QEC) to assess participants' ergonomic risk exposure and Work Ability Index (WAI) to evaluate their work ability. Demographics and other factors related with WAI were also included in the data collection. Multiple linear regression was applied to analyze the association between ergonomic exposure scores and WAI.

**Results:**

A total of 218 participants (94.8%) had valid data and consent forms. The participants' average WAI was 39.6, of which the poor and moderate WAI composed 31%. High and very high ergonomic risk exposure level was 45.9% for the neck and 21.1% for the wrist/hand. In general, WAI decreased with higher ergonomic exposure level. With adjustment of other potential risk factors, the ergonomic scores for wrist/hand and total scores for the whole body were significantly associated with the decreased WAI.

**Conclusion:**

High ergonomic risk exposure might risk in reducing work ability among young dental professionals. Intervention measures toward ergonomic risk should be taken to prevent WAI from decreasing in their early careers.

## 1 INTRODUCTION

Occupational health in dentistry has been drawing more attention in current research. Dentistry is a profession that needs both high technical skills and physical ability. A number of previous studies worldwide including China have reported a high prevalence of musculoskeletal disorders (MSDs) among dental professionals because of exposure to heavy ergonomic loading, such as awkward position, over flexed neck, rotated wrists and extended static posture.^1^, ^2^, ^3^, ^4^ It is estimated that the prevalence of general musculoskeletal pain ranges between 64% and 93%.^5^ Even worse, MSD symptoms often started as early as the student or internship phases.^6^ Dental professionals are prone to suffer from MSDs, and result in an early retirement, losing workdays, or disability.^7^

Work ability is defined as the employee's capacity to perform a job considering work demands (physical and psychological demand), health, and mental resources.^8^ It is known that maintaining and promoting work ability is important for a professional in daily practices throughout work life. Previous studies on occupational health in dentistry mostly focused on the MSDs and addressing how to combat such diseases.^9^ Very few data reported work ability or preventing work ability from declining in dental professionals,^10^ although there was some data reporting work ability in nurses and public health professionals.^11^, ^12^ To the best of our knowledge, no literature has examined the association between ergonomic risk exposure and work ability among dental professionals. Therefore, this investigation was conducted to assess the ergonomic risk exposure and work ability in dental professionals, and further determine whether high ergonomic risk exposure has an impact on work ability in their early careers.

## 2 SUBJECTS AND METHODS

### 2.1 Subjects

We carried out a cross‐sectional study in three hospitals in Guangzhou, south of China. Inclusion criteria for participants were an employing duration in dentistry of no more than 5 years and no congenital MSDs. Very few data showed the correlation between Work Ability Index (WAI) and ergonomic exposure in previous literature, so we assumed the correlation coefficient between WAI and ergonomic exposure scores to be 0.20. According to the method,^13^ the sample size needs 194 participants. Considering a 10% loss of subjects during the investigation, thus a total of 215 cases were needed. During January 2014 and May 2014, a total of 230 young dental professionals including dentists, assistants, and nurses were clustered sampled in the hospitals. We successfully recruited 218 participants with valid data and consent forms. No significant difference was found between the participants and those missed subjects on gender, age, and ergonomic exposure level distribution. Ethics approval was obtained from the ethics committee of Putian University.

### 2.2 Measurements and data collection

#### 2.2.1 Ergonomic risk exposure assessment

We applied Quick Exposure Check (QEC) to assess participants' ergonomic exposure.^14^ The QEC has been validated in the Chinese population and the intra‐ and inter‐raters reliability was also examined to be reliable.^15^, ^16^ Three interviewers who had been trained with QEC completed the field observations and interviews during participants' daily work practices. Four body parts of participants were observed and assessed for the calculation of ergonomic exposure scores and exposure level based on the algorithm, including neck, shoulders/arms, back, and wrists/hands.^14^ We calculated the total ergonomic exposure scores by adding four body parts' score and stress, vibrations, and work pace score as well. Total exposure scores were divided into four levels based on quartile.

#### 2.2.2 Work Ability Index

Work Ability Index was assessed by face to face using the WAI questionnaire. WAI has been confirmed to be a simple and reliable tool to apply in work ability assessment.^17^ It also was validated in occupational health context and applications in China.^18^ Briefly, the WAI questionnaire contains seven aspects in assessing work ability, including current work ability compared to the lifetime best, work ability in relation to the demands of the job, number of current diseases diagnosed by physician, estimated work impairment due to diseases, sick leave during the past year, and own prognosis of work ability 2 years from now and mental resources. WAI score ranges from 7 to 49 and further classified into four levels, eg, poor (7‐27), moderate (28‐37), good (38‐43), and excellent (44‐49).

Other than QEC and WAI information collection, demographics, daily physical exercises time, height/weight, and smoking and drinking habits were also included in the data collection.

### 2.3 Statistical analysis

Data analyses were centered on the association between ergonomic risk exposure and WAI while other potential confounding factors were adjusted for. Exposure score was considered as a continuous variable and further classified into four exposure levels as QEC method described.^14^ We applied multivariate linear regression to determine the associations between ergonomic risk exposure and WAI, with adjustment of gender, age, employment duration, education, marry status, Body Mass Index, smoking and drinking habits, and physical exercise time per day. Total ergonomic exposure scores and WAI scores were normally distributed among the participants (skewness = 0.472, and −0.541, respectively). WAI was used as outcome in the data analysis. We used a stepwise procedure with the criterion of entry (0.05) and removal (0.1) to select WAI‐related variables; we coded categorical variables (including physical exercise daily time) as dummy variables. Due to colinearity between different body parts of exposure scores and the total exposure scores for the whole body, we constructed two models that incorporated the exposure scores of each body part and total exposure scores with all variables meeting the criteria, respectively. *P* < .05 was assumed as a statistical significance with our study sample size. All data analyses were carried out with the SPSS version 21.0 for Windows (SPSS Inc, Chicago, IL).

## 3 RESULTS

Table 1 shows the basic demographics of participants and their work ability. Young age (27 years old), female predominant (70%), short employing duration (4 years), single status (75%), and high education level (college and above 95%) were their demographic characteristics. Seldom of them had cigarette smoking and 13% had alcohol drinking. Forty three percent of them did daily physical exercises more than half an hour. Average WAI was 39.6 for the participants, and poor/moderate WAI accounted for 31.2% (poor 1.8%, moderate 29.4%) and good/excellent for 68.8% (good 40.4%, excellent 28.4%).

**TABLE 1:** Participants' demographics and work ability

Characteristics	
Gender, n (%)	
Male	66 (30.3)
Female	152 (69.7)
Age, y, mean (SD)	27.33 (5.74)
Employment duration, y, mean (SD)	4.10 (5.37)
BMI, kg/m^2^, mean (SD)	20.56 (3.07)
Marital status, n (%)	
Single	164 (75.2)
Married or others	54 (24.8)
Educational level, n (%)	
High school	10 (4.6)
College	130 (59.6)
Graduate	78 (35.8)
Job title, n (%)	
Dental nurse/Assistant	118 (54.1)
Dentist	100 (45.9)
Work shift	
Day	212 (97.2)
Rotation	6 (2.8)
Smoking status, n (%)	
No	210 (96.3)
Yes	8 (3.7)
Drinking habit, n(%)	
No	190 (87.2)
Yes	28 (12.8)
Physical exercise time per day (min), n (%)	
<30	124 (56.9)
30‐60	72 (33.0)
>60	22 (10.1)
Work Ability Index, mean (SD)	39.1 (5.8)
Poor, n (%)	4 (1.8)
Moderate, n (%)	64 (29.4)
Good, n (%)	88 (40.4)
Excellent, n (%)	62 (28.4)

Table 2 shows ergonomic exposure scores and levels for different body parts. For neck, 45.9% of participants had high/very high exposure level, indicating the awkward posture in nearly half dental professionals. For wrist/hand, 21.1% of them had high/very high exposure level, which reflected heavy ergonomic loading during daily practices. Few participants were exposed to high or very high ergonomic level on back and shoulder/arm.

**TABLE 2:** Ergonomic exposure scores/levels for different body parts

Body parts	Neck	Shoulder/arm	Back	Wrist/hand	Total scores
Ergonomic exposure scores, mean (SD)	11.96 (3.28)	20.17 (5.87)	16.09 (5.25)	24.24 (7.51)	85.53 (21.32)
Exposure levels, n (%)					
Low	4 (1.8)	140 (64.2)	90 (41.3)	88 (40.4)	<67.75
Moderate	114 (52.3)	66 (30.3)	110 (50.5)	84 (38.5)	67.75‐82.00
High	66 (30.3)	12 (5.5)	16 (7.3)	44 (20.2)	82.00‐98.25
Very high	34 (15.6)	0	2 (0.9)	2 (0.9)	>98.25

Table 3 shows WAI changed by ergonomic exposure level. There was a declining tendency of WAI with higher ergonomic exposure level. Those with low exposure level had the highest WAI while those with very high exposure level had the lowest WAI.

**TABLE 3:** Work ability Index among different ergonomic exposure levels (mean, SD)

Exposure level	Total exposure^a^	Body parts			
		Neck	Shoulder/arm	Back	Wrist/hand
Low	41.50 (6.08)	42.50 (4.04)	39.84 (5.81)	39.83 (6.58)	40.31 (5.71)
Moderate	38.91 (5.20)	39.53 (6.28)	39.48 (5.84)	39.95 (4.94)	39.60 (6.18)
High	39.77 (5.94)	40.38 (4.08)	37.67 (6.02)	37.06 (6.39)	38.25 (5.35)
Very high	38.33 (5.76)	38.09 (7.01)	/	32.00 (1.41)	39.00 (2.83)
*P* value*	.025	.199	.403	.043	.300

^a^ Total exposure scores were categorized into four levels based on quartile.

* Statistical analyses among exposure levels using ANOVA.

Table 4 shows the association between ergonomic exposure scores and WAI while other potential factors were adjusted for. The ergonomic exposure scores for the whole body and the wrist/hand were significantly associated with decreased WAI. Physical exercise 30‐60 min/d was associated with increased WAI.

**TABLE 4:** Regression analysis for determinants of WAI

Predictors	*B* (95% CI)	Beta	*P*	VIF
Gender	−0.495 (−2.379, 1.389)	−0.039	.605	
Age	−0.008 (−0.154, 0.138)	−0.008	.912	
BMI	−0.146 (−0.446, 0.154)	−0.077	.338	
Physical exercise time per day^a^				
30‐60 min	2.444 (0.802, 4.086)	0.211	.004	
>60 min	2.068 (−1.216, 5.352)	0.089	.216	
Ergonomic exposure scores^b^				
Neck scores	−0.135 (−0.380, 0.109)	−0.076	.276	3.140
Shoulders/arms scores	−0.044 (−0.179, 0.091)	−0.044	.523	2.934
Back scores	−0.077 (−0.226, 0.071)	−0.070	.307	3.428
Wrist/hand scores	−0.135 (−0.241, −0.030)	−0.175	.012	2.166
Total exposure scores^c^	−0.053 (−0.090, −0.017)	−0.195	.004	

Abbreviations: VIF, variance inflation factor; WAI, Work Ability Index.

^a^ Included in the model as a dummy variable.

^b^ *R* ^2^ = .323 for model explanation with including each body part exposure scores, n = 218.

^c^ *R* ^2^ = .339 for model explanation with only including the total exposure scores, n = 218.

## 4 DISCUSSION

We examined the association between ergonomic risk exposure and work ability among young dental professionals in China. Our results found that ergonomic risk exposure was negatively associated with work ability, particularly wrist/hand risk exposure. This study demonstrated the impact of ergonomic risk exposure on work ability among dental professionals has occurred in their early careers.

WAI is an instrument used in clinical occupational health and research to assess work ability during health examinations and workplace surveys.^17^ Young professionals were generally thought to have high percentage of good/excellent component of work ability. In this study, the average WAI of participants was 39.1, of which 31.2% had a reduced work ability (poor and moderate). The average WAI was lower than the reference data of similar age groups among professionals in China (39.9).^18^ Meanwhile the percentage of reduced work ability was higher than the reference data (24% for the same age group).^18^ The results indicated that WAI is reduced early on in dentistry. Few studies have reported WAI in dental professionals, and none of them have provided ergonomic exposure data concurrently. Four body parts of ergonomic exposure were provided in this study. The results showed the higher exposure level the lower WAI, and further multivariate analysis confirmed the negative association with WAI. Marklund et al reported 33% of dentists had reduced work ability, which was related to poor sleep, high amount of stress, and multisite pain.^10^ A study from Brazil reported that MSDs were significantly associated with work ability in a public health institution.^11^ A recent review showed that workplace interventions did not find a high‐quality effect on work ability but a small positive effect was identified in the meta‐analysis.^18^ The workplace interventions were multilevel and individual based, eg, work arrangement, tool selection, behavior change, and exercise programs, which could be explained as surrogates of ergonomic improvements. From this point of view, ergonomic improvement might have positive association with promoting work ability, although the quality of the effect was moderate.^19^

In the nursing industry, pain from MSDs might affect work performance and result in the decline in WAI.^12^ MSDs could not only decrease work ability by interfering with the physical demand and function capacity but also affect the psychosocial aspects of work.^12^ Many epidemiological studies reported that heavy ergonomic loading caused high prevalence of MSDs, particularly in dentistry.^1^, ^2^, ^3^, ^4^ Thus, we supposed that high ergonomic risk exposure is associated with MSDs and might have further resulted in the decreased work ability. However, another study showed that a subgroup of workers with chronic musculoskeletal pain could stay at work with high work ability and performance, especially when they have high beliefs of pain self‐efficacy.^20^ This might be explained by the fact that work ability is not merely determined by individual health conditions, but work environment, including organizational environment, ergonomic friendly, or low ergonomic risk might also play a role in maintaining WAI. Another finding in our study is that a physical exercise time of 30‐60 minutes was significantly associated with increased WAI. This result is consistent with previous reports concluded that physical exercises can improve work ability.^21^, ^22^

Regarding preventing MSDs, several reviews reported evidence‐based preventive strategies for dental professionals. Most of the studies have proposed that stretching after each work session or physical strength training was effective in preventing work‐related musculoskeletal disorders (WMSDs) among dental professionals. Moderate evidence has showed that using modern ergonomic instruments and keep a neutral balance posture was beneficial to prevent WMSDs.^23^, ^24^, ^25^ Regarding improving WAI, a meta‐analysis showed workplace interventions, including individual level such as exercise or lifestyle education and multilevel factors like empowerment training or using tools to design rest breaks, had a small positive effect on work ability.^19^ However, the evidence of prevention measures was limited and even poor, which highlights the need of well‐designed, conducted, and reported randomized controlled trails, with long‐term follow‐up that assess preventive strategies for WMSDs and/or promoting WAI among dental professionals.

### 4.1 Strengths and Limitations

To the best of our knowledge, this is one of the very few studies reporting the association between ergonomic risk exposure and WAI among young dental professionals. Strengths of our study include a high response rate (95%), ergonomic exposure assessment by observations, and other potential confounding factors in the multivariate regression. The consistent negative associations between each body part exposure and WAI were not completely due to recall bias and interviewer/observation bias because three interviewers/observers did not know our study hypothesis at the time of observation and interview. Inaccuracies could have occurred during collection of information on other possible confounding factors and resulted in misclassifications, but such misclassifications were unlikely differential and thus did not significantly distort the association between ergonomic exposure and WAI.

## 5 CONCLUSION

We assessed ergonomic risk exposure and its impact on WAI among young dental professionals in China. High ergonomic risk exposure might put risk in reducing work ability even in their early professional careers. Intervention measures toward ergonomic risk should be taken to prevent WAI from decreasing in dental professionals. In addition, future extensive education programs should be launched to enhance awareness of ergonomic risk exposure and its adverse health effects, and the risk of declining work ability throughout work life.

## DISCLOSURE

*Approval of the research protocol*: The Research Ethics Committee of the Putian University Ethics Committee (no: 201901903) reviewed and approved the aims and procedures of this study. *Informed consent*: Informed consent was obtained from all individual participants included in the study. *Registry and the registration no. of the study/trial*: N/A *Animal studies*: N/A *Conflict of interest*: The authors declare that there is no conflict of interests regarding the publication of this article.

## AUTHORS' CONTRIBUTION

Dr Lin conducted the analysis and drafted the manuscript. Miss Tang, Mr Zeng, and Dr Wu performed the field survey for ergonomic risk exposure and work ability survey. Prof Xu helped to compose the idea, review the writing, and provided constructive suggestions for further refinements. All authors read the manuscript and approved to submission.
